# Integrating Events Across Levels of Consciousness

**DOI:** 10.3389/fnbeh.2013.00068

**Published:** 2013-06-14

**Authors:** Katharina Henke, Thomas P. Reber, Simone B. Duss

**Affiliations:** ^1^Division of Experimental Psychology and Neuropsychology, Department of Psychology, University of Bern, Bern, Switzerland; ^2^Center for Cognition, Learning and Memory, University of Bern, Bern, Switzerland

**Keywords:** episodic memory, unconscious, masking, subliminal, associations, flexibility, compositionality, memory systems

## Abstract

Our knowledge grows as we integrate events experienced at different points in time. We may or may not become aware of events, their integration, and their impact on our knowledge and decisions. But can we mentally integrate two events, if they are experienced at different time points and at different levels of consciousness? In this study, an event consisted of the presentation of two unrelated words. In the stream of events, half of events shared one component (“tree desk” … “desk fish”) to facilitate event integration. We manipulated the amount of time and trials that separated two corresponding events. The contents of one event were presented subliminally (invisible) and the contents of the corresponding overlapping event supraliminally (visible). Hence, event integration required the binding of contents between consciousness levels and between time points. At the final test of integration, participants judged whether two supraliminal test words (“tree fish”) fit together semantically or not. Unbeknown to participants, half of test words were episodically related through an overlap (“desk”; experimental condition) and half were not (control condition). Participants judged episodically related test words to be closer semantically than unrelated test words. This subjective decrease in the semantic distance between test words was both independent of whether the invisible event was encoded first or second in order and independent of the number of trials and the time that separated two corresponding events. Hence, conscious and unconscious memories were mentally integrated into a linked mnemonic representation.

## Introduction

No event equals another event. But events may share aspects such as a person or an item in the scene. Such commonalities between events help us to bridge events, to integrate information from events, and to make inferences that guide our choices in new situations. We are usually aware of the events that we experience. But the act of our mental integration of memories of several events and its impact on our choices and behaviors may escape our awareness. While most past experiments aimed at investigating memory for single, discrete events, there is mounting evidence that (conscious) memories of multiple events are integrated into networks, which form the basis of inference (Heckers et al., 2004; Preston et al., 2004; Smith and Squire, 2005; Shohamy and Wagner, 2008; Zeithamova and Preston, 2010; Zeithamova et al., 2012). In some experiments, integration and inference were unconscious but the encoding of events was conscious (Greene et al., 2001, 2006; Leo and Greene, 2008). In other experiments, all mental processes were unconscious, namely the processing of subliminal (invisible) events, their mental integration, and resulting inference as measured behaviorally in a test situation (Reber and Henke, 2012; Reber et al., 2012).

Neuroimaging studies in volunteers showed that the hippocampus, a brain structure that is crucial for episodic memory (Tulving, 2002; Squire, 2004), was activated when overlapping events (i.e., events with common components) were experienced and/or when inferences were made in the test situation (Heckers et al., 2004; Preston et al., 2004; Greene et al., 2006; Shohamy and Wagner, 2008; Zeithamova and Preston, 2010; Reber et al., 2012; Zeithamova et al., 2012). Importantly, the hippocampus assisted these mental operations even when they occurred outside conscious awareness (Reber et al., 2012).

A fundamental question concerns whether and how conscious and unconscious memories interact. We ask whether the mental integration of discrete events is possible if consciousness divides between events. This may happen if a first event is experienced consciously, while a second event is experienced unconsciously; or vice versa. We hypothesized that two discrete, overlapping events can be bridged through integration of memory traces across consciousness levels because evidence indicates that memories of unconsciously and consciously experienced events are laid down in the same memory system, namely the hippocampus and related cortices (Henke et al., 2003; Degonda et al., 2005; Reber et al., 2012). Hence interactions between conscious and unconscious memory traces in this study are thought to occur within the same memory system and not between different memory systems. Earlier reports of implicit-explicit interactions concerned psychologically and neuroanatomically separate memory systems that either cooperated or competed in the process of learning a material or a certain procedure (Wagner et al., 2000; Poldrack et al., 2001; Voermans et al., 2004; Moses et al., 2010). Here, we study implicit-explicit interactions within a single memory system, namely episodic memory or the medial temporal lobe memory system, whose output is either accessible or inaccessible to conscious awareness depending on the input – subliminal versus supraliminal. We expect conscious and unconscious memory representations to be integrated into a cohesive memory space that guides choices in a test situation.

**Figure 1:**
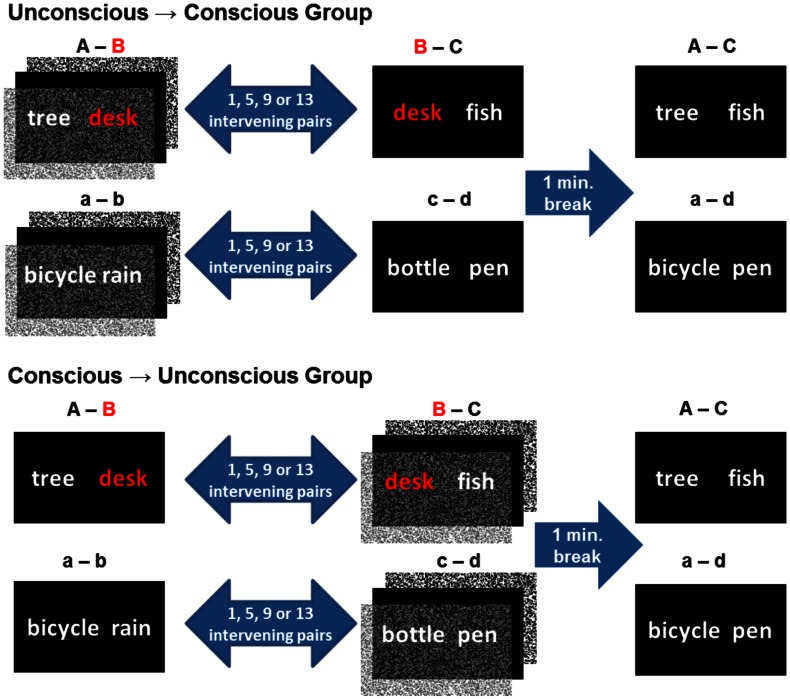
**Design**. An event consisted of the presentation of two unrelated words. Participants experienced two overlapping (experimental condition; overlap indicated in red) or non-overlapping (control condition) encoding events. The temporal distance and amount of distraction given between two corresponding overlapping and non-overlapping events was varied in four levels with 1, 5, 9, or 13 word pairs presented in-between. Depending on the group assignment, either the first or the second encoding event was presented subliminal for unconscious encoding. The subliminal presentation mode is graphically illustrated by forward and backward pattern masks. We refer to overlapping word pairs presented in the experimental condition as A–B and B–C and to non-overlapping word pairs presented in the control condition as a–b, c–d. Words in test events were presented supraliminal. A and C words from the two encoding events were re-presented at test to induce the retrieval of the common counterpart (B) in the experimental condition. In the control condition, a and d words were re-presented at test, each of which could activate its respective counterpart (b or c), but no common counterpart. Hence retrieval words given in the control condition were not episodically related by a word that was part of both encoding events.

An event was operationalized as the visual presentation of two unrelated words, such as “tree desk.” In the case of “unconscious events,” word pairs were presented subliminal, i.e., for 17 ms and flanked by pattern masks. In the case of “conscious events,” word pairs were presented supraliminal (visible). Half of word pairs shared one word, e.g., “tree desk” and “desk fish,” and were therefore overlapping (experimental condition). The other half of word pairs was non-overlapping because they shared no words (control condition) (Figure 1). To examine whether integration success is modulated by intervening time and trials, we varied the number of word pairs that intervened two overlapping word pairs by presenting 1, 5, 9, or 13 word pairs in-between. Of the two corresponding word pairs, the first was presented subliminal and the second supraliminal to half of participants, reversed for the other half of participants. We hypothesized that overlapping events would be integrated across consciousness levels irrespective of whether the first or the second event was processed consciously. Either way, a successful integration of word pairs across events and across consciousness levels presupposes that within-event associations be established. The test of integration was given 1 min following encoding. This test required participants to judge whether two supraliminal words fit together semantically or not. The two test words were either unrelated (control condition) or episodically related (e.g., “tree fish”) through two distinct events (“tree desk”… “desk fish”) that shared one word (experimental condition) (Figure 1). Words in test pairs of both conditions had been seen during encoding; one word with and the other word without consciousness. The only difference between conditions was the unconscious episodic link between test words. The mental integration of overlapping events was expected to change the speed of participants’ decisions and/or the outcome of their decisions at test (Reber and Henke, 2012; Reber et al., 2012).

## Materials and Methods

### Participants

Sixty students participated in the study (age: 19–35 years, *M* = 22.95, SD = 3.10; 40 women; six left-handers). At the point of testing, all participants had already 12 years of education. Exclusion criteria were a native language other than German, suboptimal visual acuity, a history of neurological or psychiatric disorders, and the consumption of legal or illegal drugs. Participants were kept naïve regarding the study purpose and the presentation of subliminal stimuli. We misinformed participants initially that we investigated attention and word processing alone, but participants were fully debriefed following the main experiment. The study was approved by the local ethics committee.

### Materials and design

The 384 German nouns of the main experiment were arranged to 192 semantically unrelated pairs that we presented to participants for conscious and unconscious encoding. These 192 word pairs were divided into two stimulus lists: one list was used for the experimental and the other for the control condition (96 word pairs per list/condition). Word overlaps were introduced in 48 couples of pairs (short hand: A–B, B–C; Figure 1) in list 1, which half of participants received for integrative encoding in their experimental condition. This half of participants received the 96 non-overlapping word pairs (short hand: a–b, c–d; Figure 1) of list 2 for non-integrative encoding in their control condition. The other half of participants received list 1 in the control condition and list 2 in the experimental condition. Accordingly, word overlaps were introduced in the 48 couples of pairs in list 2 for integrative encoding, while word pairs in list 1 were left non-overlapping. Importantly, all participants received the same word pairs for retrieval, counterbalanced between conditions. Hence, words in a given retrieval word pair were episodically related for half of participants (experimental condition) but unrelated for the other half of participants (control condition). Retrieval word pairs in both conditions consisted of the left-hand word of the first encoding word pair followed by the right-hand word of the corresponding second encoding word pair.

**Figure 2:**
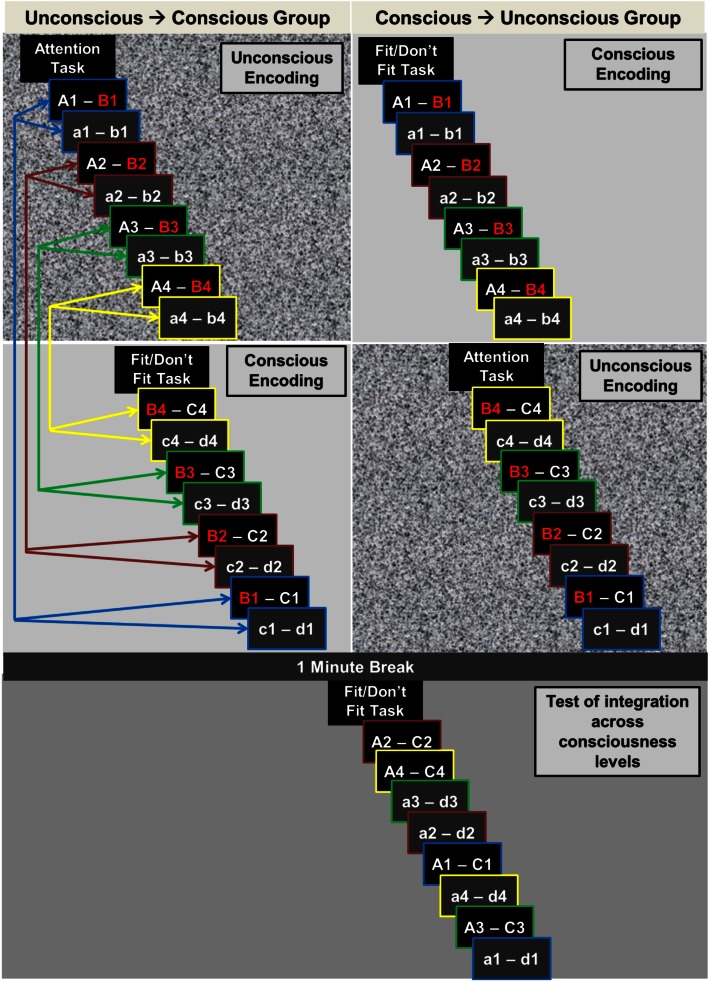
**Experimental procedure**. The experiment consisted of 12 encoding-test runs. Here, we illustrate the procedure of one run. For the unconscious → conscious group (left-hand upper panel), the first encoding word pair (A–B and a–b) was presented subliminally (illustrated by the visual noise background) and the second, corresponding encoding word pair supraliminally (B–C and c–d). This order was reversed for the conscious → unconscious group of participants (right-hand upper panel). Corresponding encoding word pairs were overlapping in the experimental condition (A–**B**, **B**–C; overlaps depicted in red) and non-overlapping in the control condition (a–b, c–d). The distance between two corresponding encoding word pairs was varied fourfold with 1, 5, 9, or 13 intervening word pairs. The four distance levels are highlighted with color-coded arrows and color-coded image-frames. The same distances applied to the procedure in the conscious → unconscious group (arrows are omitted in right-hand panel). A break of 1 min separated encoding and test. Test trials of the experimental and the control condition were presented in random order and with supraliminal duration for participants in both groups. Both in supraliminal encoding and supraliminal test trials, participants decided whether the two words of a pair fit together semantically or not. During subliminal trials, participants performed an attention task.

Stimuli of the experimental and the control condition were assigned to 12 sets that were used in 12 encoding-retrieval runs (Figure 2). Each run contained eight overlapping encoding word pairs in the experimental condition (A–B, B–C), eight non-overlapping encoding word pairs in the control condition (a–b, c–d), and four retrieval word pairs both in the experimental condition (A–C) and the control condition (a–d). The assignment of the 12 stimulus sets to runs was random. The practice run given before the main experiment was designed identical to the 12 experimental runs.

### Apparatus

The experiment took place in a dark room. A digital light processing (DLPTM) video beamer with a refresh rate of 60 Hertz projected the stimuli on a white screen positioned 2 m in front of the participant. The participant sat in a chair with his/her head fixated on a chin rest. The stimulated visual field spanned 10 (height) × 13 (width) degrees. For stimulus presentation we used the software Presentation ^®^(http://www.neurobs.com/). All responses were recorded with a standard computer mouse.

### Procedure

#### Encoding

The first encoding word pair was presented subliminal and the corresponding second word pair supraliminal for half of participants (“unconscious → conscious group”) and vice versa for the other half of participants (“conscious → unconscious group”) (see Figure 2). If word pairs appeared supraliminal (presentation duration: 3.5 s; inter-stimulus interval: 1 s), participants decided for each pair whether the two words fit together semantically or not (fit/don’t fit task). This task invokes mental comparison processes that provide for incidental paired-associative semantic encoding of words. Because words in pairs were not closely associated semantically, participants were encouraged to relax their response criterion to arrive at about 50% fit responses. Participants were informed that closely related words such as “needle – yarn” would not be presented. Instead, word pairs such as “cow – grill” would be presented. Although such words are rather remote semantically, they may still elicit a “fit” answer because beef for example is a popular sort of meat for barbecues.

When word pairs were subliminal, participants perceived a flickering stream of pattern masks and simultaneously performed an attention task on images that were embedded in the sequence of subliminal words and pattern masks. We used the masking paradigm of our previous studies on subliminal encoding (Degonda et al., 2005; Duss et al., 2011; Reber and Henke, 2011, 2012; Reber et al., 2012; see “Subliminal Stimulus Presentation and Attention Task”). An instruction slide presented before the block of eight subliminal encoding pairs and the block of eight supraliminal encoding pairs prepared participants for their up-coming task (Figure 2). Time and trials between two corresponding word pairs were manipulated; we presented 1, 5, 9, or 13 word pairs in-between two corresponding encoding word pairs (Figure 2). Word pairs were randomly assigned to interval levels. Following encoding, participants took a 1-min break that served as a mini consolidation phase.

#### Retrieval

To test for relational integration, we presented four retrieval pairs in each condition. The order of these eight pairs was randomly generated for each participant and each run. Retrieval word pairs were presented in the same fashion (presentation duration: 3.5 s; inter-stimulus interval: 1 s) and with the same task (fit/don’t fit task) as supraliminal encoding word pairs.

#### Subliminal stimulus presentation and attention task

We used the masking paradigm of our previous studies on subliminal encoding (Degonda et al., 2005; Duss et al., 2011; Reber and Henke, 2011, 2012; Reber et al., 2012). Each encoding word pair was flashed 12 times for 17 ms within a time window of 6 s that constituted one encoding trial. Word pairs were flanked by black and white dot pattern masks that were presented for 183 ms. In this stream of subliminal word pairs and pattern masks, we embedded an attention task for participants to stay focused and direct gaze at the screen center. The attention task required participants to fixate a repeatedly flashed central fixation cross and to indicate when the fixation cross was replaced by a horizontal or vertical line segment (push left or right key, respectively). The fixation cross was presented for 233 ms at a rate of one Hertz. The fixation cross was replaced only once by a line segment at a random time point within the 6-s time window that constituted a trial.

### Test of awareness

Following the main experiment, participants underwent a structured interview to find out whether they had noticed or suspected the presence of subliminal stimuli in the experiment. Next, participants were informed of subliminal word pairs in the experiment. Finally, they took an objective awareness test to assess their ability to consciously discern subliminal word pairs or fragments thereof. Participants were instructed to try to discern a subliminal word pair and to match it to a subsequently presented identical or different word pair on a trial-by-trial basis. While instructions in the main experiment were indirect not alluding to subliminal word pairs, instructions in the test of awareness were direct. Direct tests are predominantly sensitive to conscious rather than unconscious processes (Reingold and Merikle, 1988; Snodgrass and Shevrin, 2006) and therefore yield a measure of conscious access to subliminal stimuli. Indirect tests are predominantly sensitive to unconscious rather than conscious processes. We aimed at indirectly measured effects of unconscious processing with no directly measured effects of conscious word detection (Reingold and Merikle, 1988; Greenwald et al., 1995). The test of awareness was different for the two experimental groups because subliminal words (B words in B–C pairs) were primed by previously perceived supraliminal words (A–B pairs) in the conscious → unconscious group but not the unconscious → conscious group.

#### Test of awareness for the unconscious → conscious group

**Figure 3:**
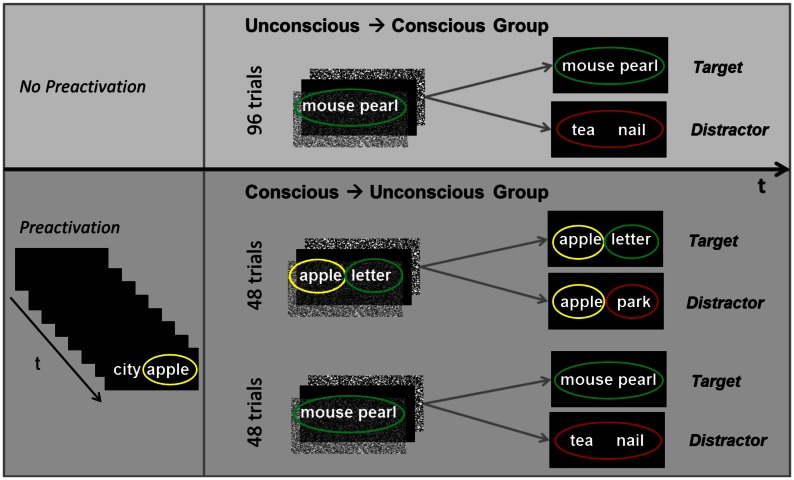
**Design of the test of awareness**. Following the main experiment, participants took an objective awareness test to assess their ability to consciously discern subliminal word pairs or fragments thereof. Participants were instructed to try to discern a subliminal word pair and to match it to a subsequently presented identical (target) or different (distractor) word pair on a trial-by-trial basis. The test of awareness was different for the two experimental groups because subliminal words (B words in B–C pairs) were primed by previously perceived supraliminal words (A–B pairs) in the conscious → unconscious group (bottom panel) but not the unconscious → conscious group (top panel). *t*, time.

The test of awareness included 96 trials. Each trial consisted of the subliminal presentation of one word pair followed by the supraliminal presentation of either the same or a different word pair (Figure 3). The masking paradigm and psychophysical conditions applied in the main experiment were again applied in the test of awareness. Participants were instructed to attend to the subliminal presentation of a word pair, while doing the attention task, and then to indicate by button press whether the subsequently presented visible word pair corresponded to the subliminal word pair (target) or not (distractor). Supraliminal targets and distractors stayed on the screen till participants responded. The probability of targets was 50%.

#### Test of awareness for the conscious → unconscious group

This awareness test included 96 trials as well, but these trials were divided into six runs that each contained 16 subliminal-supraliminal test trials. As in this group’s main experiment, each run started off with the supraliminal presentation of eight A–B word pairs for participants to decide whether words in a pair fit together or not (fit/don’t fit task). This preactivation was followed by the 16 subliminal-supraliminal test trials. The presentation order of the 16 trials was randomized. Eight of the 16 trials contained subliminal pairs with a primed B word (B–C trials) and eight trials contained subliminal pairs with unprimed words (Figure 3). Each of the subliminally presented word pairs was immediately followed by a supraliminal word pair (target or distractor) for participants to indicate by button press whether the visible word pair was identical to the subliminal word pair or not. In trials where subliminal word pairs were unprimed, targets were equal to subliminal words and distracters differed (as for the unconscious → conscious group). However, in trials where the first word in a subliminal pair was primed (e.g., apple), targets were equal to subliminal words, while distractors were composed of the same first word (e.g., apple) plus a second new word (e.g., park) (Figure 3). Hence, both targets and distractors contained the primed word (e.g., apple). This circumstance allowed us to test only for the perceptual processing of subliminal new words that were presented besides the primed words in pairs. Hence, with this procedure we could not test for a perceptual advantage of the primed words contained in subliminal pairs. Even if only the targets, but not the distractors, would contain a primed word (e.g., apple), participants’ selections would still fail to indicate whether the subliminal processing of primed words is facilitated because the absence versus presence of a previously seen (during preactivation) and probably remembered word would skew selections in favor of targets.

### Statistical analyses

For the analysis of performance accuracy at test, we computed the rate of each participant’s fit responses to A–C pairs (experimental condition) and to a–d pairs (control condition) by dividing the total of fit responses by the respective total of given responses. The rate of fit responses was also computed for each encoding interval (1, 5, 9, or 13 word pairs between two corresponding encoding word pairs; see Figure 2). Trials with RTs below 500 ms were excluded. Rates of fit responses were analyzed in an ANOVA with the two within-subjects factors Condition (experimental versus control condition) and Encoding Interval (1, 5, 9, or 13 intervening word pairs) and the between-subjects factor Encoding Order (unconscious → conscious versus conscious → unconscious). For the analysis of reaction latency, RTs of fit and don’t fit responses were *z*-transformed with respect to the RT distribution of each participant. RTs with *z*-values deviating more than 2 SDs from a participant’s mean were excluded. We computed each participant’s mean RT for A–C pairs (experimental condition) and a–d pairs (control condition) and per encoding interval. Mean RTs were analyzed in an ANOVA that included the same factors as the ANOVA used for the accuracy data (rate of fit responses).

We also computed mean RTs of fit and don’t fit responses to supraliminal encoding word pairs. For participants of the conscious → unconscious group, supraliminal encoding word pairs were A–B and a–b word pairs. For participants of the unconscious → conscious group, supraliminal encoding word pairs were B–C and c–d word pairs. Differences in mean RT between conditions were tested for significance with a paired-samples *t*-test.

## Results

### Test of awareness: Performance at chance level

As in the main experiment, trials with RTs below 500 ms were excluded from the analysis. Rates of correct answers (hits plus correct rejections) in the test of awareness deviated from chance performance (0.50) neither in the unconscious → conscious group [*M* ± SE = 0.51 ± 0.01; *t*(29) = 0.969, *p* = 0.341; 96 trials] nor the conscious → unconscious group [*M* ± SE = 0.50 ± 0.01; *t*(29) = −0.407, *p* = 0.687; 96 trials]. Discrimination performance in the latter group remained at chance level, when preactivation trials [*M* ± SE = 0.49 ± 0.01; *t*(29) = −0.601, *p* = 0.553] and trials without preactivation [*M* ± SE = 0.50 ± 0.01; *t*(29) = 0.158, *p* = 0.876] were analyzed separately.

Binomial tests computed for every participant revealed that two of the 60 participants yielded a relatively good discrimination performance that reached a probability of 10% or lower (one-tailed) to be obtained by chance alone. Furthermore, one participant reported to have consciously perceived subliminal letters during the main experiment. However, this person’s performance in the test of awareness was at chance level. We decided to exclude these three participants from the analysis of the data from the main experiment. Accordingly, 28 participants remained in the unconscious → conscious group and 29 in the conscious → unconscious group.

### Conscious and unconscious encoding of events

#### Good accuracy on the attention task

Performance accuracy on the attention task given during subliminal trials was good and did not differ between the two experimental groups [*t*(37.956) = −0.982, *p* = 0.332, adjusted degrees of freedom because equality of variances was not assumed]. The unconscious → conscious group yielded a hit rate of 0.88 ± 0.02 (*M* ± SE) and the conscious → unconscious group of 0.90 ± 0.01 (*M* ± SE).

#### Subliminal B words in A–B pairs primed supraliminal B words in B–C pairs

As a consequence of the encoding of subliminal A–B pairs in the unconscious → conscious group, we expected a facilitated processing (priming) in the experimental condition, where B words were repeated in supraliminal B–C pairs. A premise of B word priming is a compositional rather than unitized representation of B words in A–B and B–C pairs. A compositional mental representation allows both for the reactivation of each individual part in a representation (A; B) and the reactivation of the complete representation (A–B). A unitized mental representation, however, would require the repeated visual presentation of the complete initial word pair (A–B), not just the B word, to trigger a reactivation of the previously formed A–B representation. Our data spoke for a compositional representation of subliminal A–B pairs. Responses to supraliminal B–C pairs were faster (*M* ± SE = 2106 ± 71 ms) than responses to supraliminal c–d pairs (*M* ± SE = 2163 ± 78 ms) [*t*(27) = 3.502, *p* = 0.002, *r*^2^ = 0.312]. The absence of a corresponding difference in the processing speed of supraliminal A–B (*M* ± SE = 2138 ± 68 ms) versus supraliminal a–b pairs (*M* ± SE = 2147 ± 65 ms) [*t*(28) = 0.440, *p* = 0.663] in the conscious → unconscious group substantiates the interpretation in terms of priming.

### Test performance: Successful integration of events

**Figure 4:**
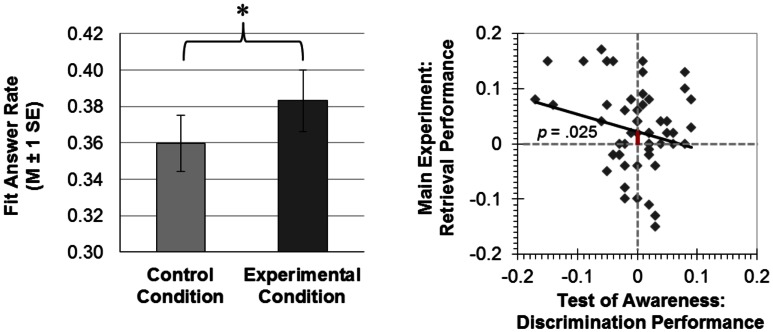
**Results**. Left-hand panel: at the test of integration across consciousness levels, participants gave more fit answers in the experimental than the control condition (*p < 0.05; *M*, mean; SE, standard error of the mean). Subliminal and supraliminal encoding word pairs had shared a word in the experimental condition, which lent these word pairs to integration that manifested in biased semantic decisions at test (more fit responses). Right-hand panel: using the regression method of Greenwald et al. (1995), we regressed the discrimination performance in the test of awareness [(hits + correct rejections) − (false alarms + misses)] onto the rate of fit responses given to episodically related (experimental condition) minus unrelated (control condition) test words. The *y*-axis intercept was significantly greater than zero. This indicates that the integration of events across consciousness levels was significant, when conscious discrimination performance was zero.

We computed an ANOVA with the two within-subjects factors Condition and Encoding Interval as well as the between-subjects factor Encoding Order. The dependent variable was the rate of fit responses given at test. This ANOVA yielded a significant main effect of Condition (Figure 4). Participants gave more fit responses to episodically related A–C pairs (*M* ± SE = 0.38 ± 0.02) versus unrelated a–d pairs (*M* ± SE = 0.36 ± 0.02), *F*(1,55) = 5.310, *p* = 0.025, $\eta_{\text{partial}}^{2}=0.088$. This effect of Condition interacted neither with Encoding Interval [*F*(3,165) = 0.011, *p* = 0.999] nor with Encoding Order [*F*(1,55) = 0.263, *p* = 0.610]. There was no main effect of Encoding Interval [*F*(3,165) = 0.234, *p* = 0.872], no significant interaction of Encoding Interval with Encoding Order [*F*(3,165) = 1.156, *p* = 0.328], and no three-way interaction of Condition with Encoding Interval and Encoding Order [*F*(3,165) = 0.379, *p* = 0.768]. The means (±SE) of the rate of fit responses were almost identical between encoding intervals (1 pair, 5 pairs, 9 pairs, 13 pairs) both for the experimental condition (*M*_1pair_ = 0.38 ± 0.03; *M*_5pairs_ = 0.38 ± 0.02; *M*_9pairs_ = 0.38 ± 0.02; *M*_13pairs_ = 0.40 ± 0.02) and the control condition (*M*_1pair_ = 0.36 ± 0.02; *M*_5pairs_ = 0.36 ± 0.02; *M*_9pairs_ = 0.36 ± 0.02; *M*_13pairs_ = 0.37 ± 0.03). However, the between-subjects factor Encoding Order reached significance [*F*(1,55) = 5.294, *p* = 0.025, $\eta_{\text{partial}}^{2}=0.088$]. A potential reason for this main effect might be the unconscious → conscious group’s criterion for generating fit responses, which might be looser yielding more fit responses (*M* ± SE = 0.41 ± 0.02) than the conscious → unconscious group (*M* ± SE = 0.34 ± 0.02). In support of this interpretation, the unconscious → conscious group also generated more fit responses during supraliminal encoding (B–C and c–d pairs; *M* ± SE = 0.45 ± 0.02) than the conscious → unconscious group (A–B and a–b pairs; *M* ± SE = 0.36 ± 0.02) [*t*(52.605) = 3.256, *p* = 0.002, *r*^2^ = 0.167, adjusted degrees of freedom because equality of variances was not assumed].

Reaction latencies at test were comparable between the experimental (A–C; *M* ± SE = 1942 ± 46 ms) and the control condition [a–d; *M* ± SE = 1952 ± 45 ms; *t*(56) = 0.854, *p* = 0.397]. This absence of an integration effect on reaction speed was also reflected in the non-significance of all results of an ANOVA that included the same factors as the ANOVA on the rates of fit responses (all *F*s < 2.469, all *p*s > 0.122). Hence, the mental integration of events across levels of consciousness influenced the type but not the speed of responses given at test.

### Conscious word discrimination did not assist the unconscious integration of events

Using the regression method described by Greenwald et al. (1995), we found that discrimination performance in the test of awareness [(hits + correct rejections) − (false alarms + misses)] was not significantly associated with the increase in the rate of fit responses given to episodically related versus unrelated test words [*B* = −0.314 ± 0.184, β = −0.224, *t*(55) = −1.705, *p* = 0.094; *N* = 57]. However, the *y*-axis intercept in this regression was significantly larger than zero [intercept = 0.023 ± 0.010; *t*(55) = 2.309, *p* = 0.025, *r*^2^ = 0.088]. This indicates that the unconscious integration of events was significant, when discrimination performance in the test of awareness was zero (Figure 4).

## Discussion

We asked whether the mental integration of discontiguous events is possible if consciousness divides between events because one event is experienced consciously and the other unconsciously. An event was operationalized as the visual presentation of two unrelated words that were either presented supraliminal for conscious inspection or subliminal for unconscious processing. At the test of integration, we presented a word from the unconscious event besides a word from the corresponding conscious event in both the experimental and the control condition. These test words were semantically unrelated. But test words in the experimental condition were episodically related by a word (not present in the test trial) that had been the counterpart of each of the test words drawn from the two encoding events. Test words in the control condition were episodically unrelated. Episodically related versus unrelated test words were more often judged as closely related semantically. It thus appears that episodic associations intruded into judgments of semantic distance leading to more fit responses in the experimental versus the control condition. This effect is known from studies with supraliminal stimulus presentations: two unrelated words that had been presented in the same encoding context and were episodically (but not semantically) related, appeared closer semantically than words that had not been presented in the same encoding context; or they appeared equally close as words that were related semantically (McKoon and Ratcliff, 1979, 1986; Dosher and Rosedale, 1991; Patterson et al., 2009; Coane and Balota, 2011). This line of research suggests that connections between mental representations or between nods in the semantic network, which have been co-activated in the same encoding context, acquire a greater linkage strength leading to the impression of stronger conceptual relatedness. The co-occurrence of concepts in naturalistic events is indeed one way how the semantic system may be dynamically (re)organized throughout life (Coane and Balota, 2011). Although test words in the present experimental condition had not occurred in the same encoding context but in two different encoding contexts, they were linked by a third word that was presented in both encoding contexts. This mediated linkage between test words had apparently sufficed to decrease the perceived semantic distance between test words. Remarkably, this was possible with one of the two events processed outside consciousness.

Do these modifications in participants’ semantic systems result from plastic chances in the neocortex alone or from additional plastic changes in the medial temporal lobe, particularly the hippocampus? The hippocampus is ordinarily assisting the rapid encoding of events by association formation between co-activated areas of the neocortex (Teyler and DiScenna, 1986; Treves and Rolls, 1994). Our task structure and results speak to a hippocampal role because encoding was rapid (one-trial) and memory representations must have been compositional and flexible (Cohen and Eichenbaum, 1993; Henke, 2010). There was no neuroimaging in the present study but earlier neuroimaging studies give a clue. Reber et al. (2012) used a similar design but presented all encoding events subliminally. There was hippocampal activation during the unconscious encoding of overlapping events and during judgments of semantic distance made on episodically related word pairs presented at test. Moreover, hippocampal activity measured during the encoding of overlapping subliminal events predicted judgments of semantic distance at test. The hippocampus was also activated in studies using supraliminal stimuli, either at the time when overlapping events were encoded or when inferences were made at test (Heckers et al., 2004; Preston et al., 2004; Greene et al., 2006; Shohamy and Wagner, 2008; Zeithamova and Preston, 2010; Zeithamova et al., 2012). Because the hippocampus played a role both in studies of inference that used supraliminal encoding events and in a study that used subliminal encoding events, we assume that the hippocampus is also engaged when encoding events were both conscious and unconscious. Increasing evidence suggests that the hippocampus engages in all tasks that require the rapid encoding of new, compositional, and flexible associations irrespective of the level of consciousness of stimulus processing (Henke, 2010).

The absence of an effect of time/stimuli between two overlapping encoding events on test performance replicates our previous result of the successful integration of two discrete subliminal events, which was also unaffected by intervening time/stimuli (Reber and Henke, 2012). The lack of a temporal distance effect in these two experiments is intriguing given previous evidence that temporal contiguity influences the retrieval from episodic memory (Temporal Context Model; Howard and Kahana, 1999; Howard et al., 2005). Core assumptions of the Temporal Context Model entail context-representations that change gradually over time and that act as retrieval cues for the items learned in the same contexts. Because items that are learned in close temporal proximity have similar contexts, they are more likely to be recalled (Howard and Kahana, 1999). If an encoding item is shown twice to boost learning, the context of its second presentation will resemble the context of its first presentation due to its presence at both moments in time. In our study, B words must have induced contiguity between the two overlapping encoding events A–B and B–C. The presence of B words in both encoding events may have rendered the two events more independent of their temporal (dis)contiguity (Howard et al., 2005). In other words, the configurational contiguity induced by the presence of B words in both overlapping events may have anchored and linked the two events to the extent of neutralizing potential adverse effects of temporal discontiguity (and interfering information) presented between overlapping events (Howard et al., 2005).

The current design does not allow pinning down the time point at which the integration of conscious and unconscious memories took place – at encoding or test. Either way, representations of unconscious events must have been solid and stable over time because test performance was not modulated by whether the unconscious event was preceding or following the corresponding conscious event. Hence, conscious and unconscious memory traces could be integrated independently of the order, in which the memory traces were formed. If integration of memory traces occurred at test, it may have been advantageous to encode the conscious event first because consciously acquired memories are stronger and decay not as rapidly as unconsciously acquired memories; after all, the time interval between the first event and the final test was as long as 170 s. Under the assumption that memories are integrated at retrieval, the lack of an order effect means that conscious and unconscious memories outlasted this time period equally well. If participants integrated memories already when the second encoding event was presented, the first memory trace had to outlast 7–72 s, which was certainly the case for both unconscious and conscious memories. The absence of an order effect additionally suggests that it was not easier to link an unconscious (weak) representation to a pre-existing conscious (strong) representation than vice versa. Hence, for these time intervals, the lack of an order effect suggests that unconscious memories were on a par with conscious memories regarding their endurance and flexibility.

We had hypothesized that conscious and unconscious memory representations could be integrated into a cohesive memory space assuming that the same memory system supports the encoding of subliminal and supraliminal events (Henke et al., 2003; Degonda et al., 2005; Reber et al., 2012). Yet, such harmonious interactions between explicit and implicit memory may not have been expected given past evidence of dissociations and competing interactions between implicit and explicit memory (Wagner et al., 2000; Poldrack et al., 2001). Such adverse interactions concerned separate memory systems (hippocampal interactions with other structures) that competed in the process of learning the same kind of material or procedure. Our study, however, concerned the encoding of distinct events by the same memory system that works at two different levels of intensity – conscious and unconscious. Furthermore, the information acquired in the first and the second overlapping event was neither identical nor conflicting (as in Degonda et al., 2005) but additive, which may have contributed to the smooth integration of information across consciousness levels.

Given that events can be encoded with and without consciousness by way of the hippocampus and related cortices (Henke et al., 2003; Degonda et al., 2005; Henke, 2010; Reber et al., 2012), the organization of consciously and unconsciously acquired information into a single, cohesive hippocampal memory space appears more economic than the organization of information in two discrete hippocampal memory spaces divided by consciousness. Linked episodic knowledge – conscious or unconscious – informs and guides us better through life than episodic knowledge segmented into levels of representation. Moreover, the level of representation of episodic knowledge is dynamic and shifts over time. A single hippocampal memory space maintains the stability and coherence of its organization when episodic memories shift from unconscious to conscious (Fischer et al., 2006; Drosopoulos et al., 2011; Yordanova et al., 2012) or from conscious to unconscious in the course of consolidation (Saletin et al., 2011).

## Conflict of Interest Statement

The authors declare that the research was conducted in the absence of any commercial or financial relationships that could be construed as a potential conflict of interest.
